# Sampling Rate Effects on Resting State fMRI Metrics

**DOI:** 10.3389/fnins.2019.00279

**Published:** 2019-04-02

**Authors:** Niko Huotari, Lauri Raitamaa, Heta Helakari, Janne Kananen, Ville Raatikainen, Aleksi Rasila, Timo Tuovinen, Jussi Kantola, Viola Borchardt, Vesa J. Kiviniemi, Vesa O. Korhonen

**Affiliations:** ^1^Oulu Functional NeuroImaging Group, Research Unit of Medical Imaging, Physics and Technology, University of Oulu, Oulu, Finland; ^2^Department of Diagnostic Radiology, Medical Research Center, Oulu University Hospital, Oulu, Finland

**Keywords:** resting state, magnetic resonance encephalography, aliasing, pulsations, quasi-periodic patterns

## Abstract

Low image sampling rates used in resting state functional magnetic resonance imaging (rs-fMRI) may cause aliasing of the cardiorespiratory pulsations over the very low frequency (VLF) BOLD signal fluctuations which reflects to functional connectivity (FC). In this study, we examine the effect of sampling rate on currently used rs-fMRI FC metrics. Ultra-fast fMRI magnetic resonance encephalography (MREG) data, sampled with TR 0.1 s, was downsampled to different subsampled repetition times (sTR, range 0.3–3 s) for comparisons. Echo planar k-space sampling (TR 2.15 s) and interleaved slice collection schemes were also compared against the 3D single shot trajectory at 2.2 s sTR. The quantified connectivity metrics included stationary spatial, time, and frequency domains, as well as dynamic analyses. Time domain methods included analyses of seed-based functional connectivity, regional homogeneity (ReHo), coefficient of variation, and spatial domain group level probabilistic independent component analysis (ICA). In frequency domain analyses, we examined fractional and amplitude of low frequency fluctuations. Aliasing effects were spatially and spectrally analyzed by comparing VLF (0.01–0.1 Hz), respiratory (0.12–0.35 Hz) and cardiac power (0.9–1.3 Hz) FFT maps at different sTRs. Quasi-periodic pattern (QPP) of VLF events were analyzed for effects on dynamic FC methods. The results in conventional time and spatial domain analyses remained virtually unchanged by the different sampling rates. In frequency domain, the aliasing occurred mainly in higher sTR (1–2 s) where cardiac power aliases over respiratory power. The VLF power maps suffered minimally from increasing sTRs. Interleaved data reconstruction induced lower ReHo compared to 3D sampling (*p* < 0.001). Gradient recalled echo-planar imaging (EPI BOLD) data produced both better and worse metrics. In QPP analyses, the repeatability of the VLF pulse detection becomes linearly reduced with increasing sTR. In conclusion, the conventional resting state metrics (e.g., FC, ICA) were not markedly affected by different TRs (0.1–3 s). However, cardiorespiratory signals showed strongest aliasing in central brain regions in sTR 1–2 s. Pulsatile QPP and other dynamic analyses benefit linearly from short TR scanning.

## Introduction

In 1995 Biswal and co-workers discovered functional connectivity (FC) in resting state blood oxygen level dependent (BOLD) signal of motor cortices by their continuous very low frequency fluctuations (VLF < 0.1 Hz) (Biswal et al., 1995). Initially, the VLF phenomena were linked to existing literature on low frequency physiological phenomena like vasomotor waves (Kiviniemi et al., 2000). The current view is that spontaneous neuronal activity avalanches synchronize brain activity in functionally connected areas and become visible in hemodynamic signals after a delay of few seconds (Liu and Duyn, 2013; Palva et al., 2013; Keilholz, 2014; Ma et al., 2016; Liu et al., 2018). The spread of such avalanches can be depicted by novel techniques such as inverse imaging (INI) and magnetic resonance encephalography (MREG), that sample functional magnetic resonance imaging (fMRI) data with short repetition times (TR) (Lin et al., 2012, 2018; Assländer et al., 2013; Rajna et al., 2015).

In addition to neuronal activity that is coupled to hemodynamics, the BOLD signal reflects multiple other signal sources, such as motion, physiological pulsations and technical artifacts. The physiological factors like cardiorespiratory changes modulate the BOLD signal and can mask the neuronally driven VLF activity in resting state BOLD signals (Wise et al., 2004; Birn et al., 2006). As the BOLD signal reflects blood oxygen level, also direct effects of the cardiorespiratory pulses themselves can be detected in fMRI data (Shmueli et al., 2007; Chang and Glover, 2009, 2010).

Previously, the research of cardiorespiratory brain pulsations has not gained as much interest because they have been deemed as noise. However, there is increasing evidence showing that these physiological signals or the “noise” it produces in TR BOLD data with long TR, can be used to measure disease-specific changes in patient groups (Makedonov et al., 2013; Tuovinen et al., 2017). This is strongly supported by the recent discovery of the glymphatic brain tissue clearance mechanism where the cardiovascular pulsations have been shown to drive the glymphatic brain clearance (Iliff et al., 2012; Nedergaard, 2013; Jessen et al., 2015). The short TR in 3D MREG can critically sample the spread of cardiovascular ~1 Hz and respiratory ~0.3 Hz pulsations and separate them from VLF (< 0.1 Hz) quasi-periodic patterns (QPPs) (Kiviniemi et al., 2016).

The extent to which faster physiological pulsations alias over VLF BOLD signal, has been a prevailing uncertainty in BOLD fMRI. Since cardiac frequencies can be faster than 2 Hz, especially in animals and children, the critical sampling rate should be >4 Hz according to the Nyquist theorem, i.e., TR <0.25 s. However, most often the fMRI TRs are >0.4 s and therefore the data cannot critically sample faster cardiac signals (Liu, 2016). Consequently, aliasing between cardiac and the VLFs occur and may alter FC metrics. Additionally, the cardiorespiratory rhythms and their pressure modulations and physiological autonomic nervous system mediated counter-regulations, local vasomotor waves induce heart rate variability, which differ between patients and controls (van der Kooy et al., 2006; Thayer et al., 2010) causing yet another confounding factor in measures of FC that may require faster sampling.

Early literature on the sampling rate on FC measures usually utilizes single slice data that suffers from out of plane motion and other registration problems (Purdon and Weisskoff, 1998; Peltier et al., 2003; Kiviniemi et al., 2005). Recent studies on the fMRI sampling rate effects on resting-state FC has shown surprisingly small effects (Golestani et al., 2017; Demetriou et al., 2018). However, that and some other recent studies have usually been limited to < 4 Hz sampling rates for whole brain coverage (Cordes et al., 2014; Liu, 2016; Golestani et al., 2017; Chen et al., 2019) with different type of signal simulations extending below the critical 4 Hz limit.

However, more and more critically sampled 3D whole brain fMRI data has started to emerge, such as 0.136 sec TR VEPI (Posse et al., 2013), 100 ms MREG (Assländer et al., 2013; Lee et al., 2013; Kiviniemi et al., 2016; Raitamaa et al., 2018), 50 ms GIN (Boyacioglu and Barth, 2013) and currently leading 25 ms 3D whole brain scan INI (Chang et al., 2013b). This critically sampled data shows robust novel phenomena of the human brain physiology, such as propagating cardiorespiratory pulsations that both interact and modulate each other, depending on their anatomical proximity to pulsation sources (Posse et al., 2013; Kiviniemi et al., 2016; Raitamaa et al., 2018). Based on our observations, these novel signal changes cannot be comprehensively simulated due to their complex spatiotemporal pattern that is dynamically changing.

Therefore, in this study we used real critically sampled 0.1 s TR 3D single shot MREG data to explore how different sampling rates affect the results of the most commonly used resting state fMRI analysis tools. The 0.1 s TR MREG signal was downsampled to higher TRs ranging from 0.3 to 3 s. The use of subsampled TR (sTR) removes the confounding factors of imaging different TR values in separate scans and/or individuals, enabling identical physiological status and technological noise structure for comparing different sTRs. The single shot 3D data sampling scheme was further compared with interleaved slice sampling (INT) variant of the MREG data, and, with conventional interleaved gradient recalled echo-planar BOLD imaging (EPI BOLD).

The hypothesis was that the faster sTR produces both spatially more accurate brain maps and more accurate time series without aliasing of physiological pulsations. The quantified connectivity metrics included stationary spatial, time, and frequency domains, as well as dynamic analyses. In addition, the effects of aliasing were evaluated.

## Materials and Methods

### Participants

Ten healthy subjects (8 males, 23.8 ± 2.1 years old) were placed in the MRI scanner and asked to lay still and keep their eyes open and fixated on a cross on the screen while thinking of nothing particular (eyes open, resting state). Ear plugs were used to reduce scanner noise. Cushions were placed beside ears to restrict movement and to further reduce scanner noise. MREG (5 min) and EPI BOLD (5 min) sequences were scanned in said order. Written informed consent was obtained from each subject prior to scanning, in accordance with the Helsinki declaration. The study protocol was approved by the regional Ethical committee of Northern Ostrobothnia Hospital District in Oulu University Hospital.

### Data Acquisition and Preprocessing

Subjects were scanned using Siemens 3T SKYRA scanner with 32-channel head coil. Additional cardiorespiratory data were collected using MRI-compatible multimodal neuroimaging system (Korhonen et al., 2014). MREG is a 3D single shot stack of spirals (SOS) sequence that under-samples k-space to reach a sampling rate of 10 Hz allowing critical imaging of physiological pulsations (Assländer et al., 2013). The SOS gathers k-space in 60 ms with spiral in/out repeating in every other turn continuously in positive z-direction to minimize the air-sinus off-resonance artifact (for more details, c.f. Assländer et al., 2013). The point spread function of the SOS-sequence is 3 mm with minimized off-resonance effects compared to other k-space undersampling strategies like concentric shells and spokes (Zahneisen et al., 2012; Assländer et al., 2013). Scanning parameters (TR = 100 ms, TE = 36 ms, flip angle = 5°, 3D matrix = 64^3^, FOV = 192 mm) enabled scanning of the whole brain in 10 Hz with voxel size of 3 × 3 × 3 mm^3^. Conventional EPI BOLD scans were collected from the same subjects (TR = 2,150 ms, TE = 28 ms, flip angle = 15°, voxel size = 3 × 3 × 3 mm, matrix size 64^*^64, 45 slices = 47 ms in plane readout). In both methods, we used relatively low flip angles to minimize specific absorption rate (SAR), spin history effects, physiological pulsations and radio frequency (RF) artifacts in EEG in comparison to default flip angles (Gonzalez-Castillo et al., 2011; Assländer et al., 2013).

**Figure 1:**
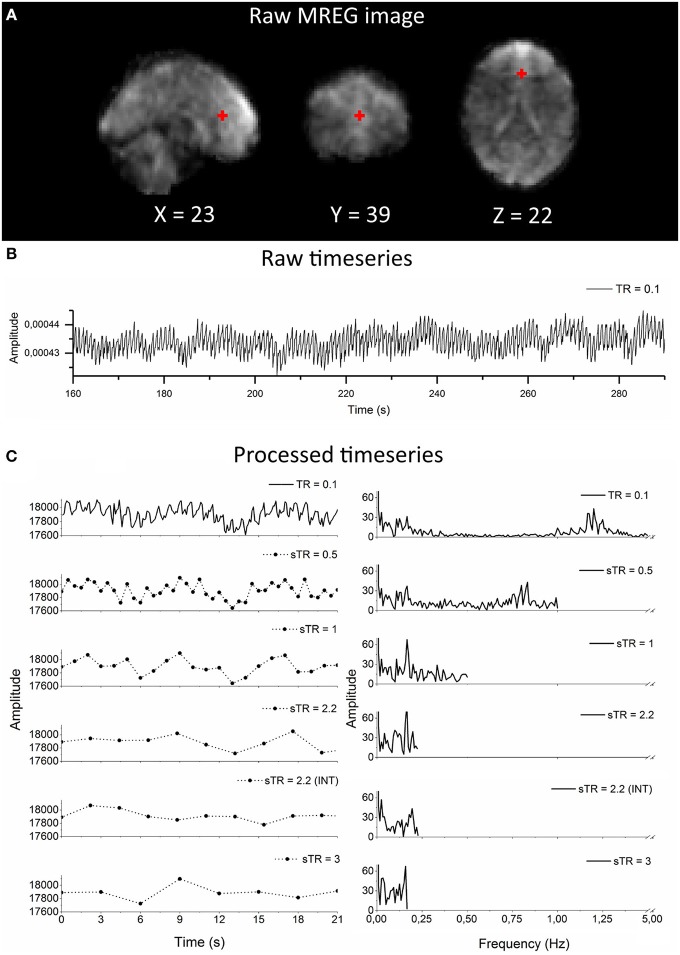
Raw magnetic resonance encephalography (MREG) data with repetition time (TR = 0.1 s) and the subsampling scheme using an example arterial signal ([−2 30 16] mm in Montreal Neurological Institutional (MNI) space). **(A)** Raw spatial MREG image. **(B)** Raw timeseries of the arterial signal. **(C)** Processed and subsampled arterial signal and corresponding frequency amplitude spectra. Please note the aliased cardiac peak in the sTR 1–3 s in the amplitude spectra at ~0.2 Hz.

A reference image for MREG was acquired with a multi slice double gradient echo sequence with TR = 593 ms, TE = 2.46/4.92 ms, flip angle = 50°, dwell time = 4.9 us, FOV = 192 mm. The reference and raw data from the MREG sequence were transferred offline to a computing grid and reconstructed using the MATLAB tool provided with the sequence. The tool allows for a choice between several parameters for regularized reconstruction (Hugger et al., 2011); we selected L2-norm with finite difference operator (called “Total Variation” in the tool) and the regularization parameter was reduced to lambda = 0.15 from default 0.2 in order to obtain higher signal-to-noise ratio (SNR) images. Conjugate gradient optimization was also performed for 35 iterations for more robust convergence of images, c.f. Figure 1. Coil sensitivities were estimated from the reference image with the adaptive method, and dynamic off-resonance correction in k-space was used to minimize respiration and other motion related off-resonance artifacts from the data (Pfeuffer et al., 2002; Zahneisen et al., 2014). Anatomical 3D MPRAGE (TR = 1,900 ms, TE = 2.49 ms, TI = 900 ms, flip angle = 9°, FOV = 240 mm, 0.9 mm cubic voxel) images were used to register both MREG and EPI BOLD data into Montreal Neurological Institute (MNI) space.

Both, EPI BOLD and MREG data were preprocessed with a typical FSL pipeline (Jenkinson et al., 2012). The data were high-pass filtered with cut-off frequency of 0.008 Hz (125 s). T1-relaxation effects were minimized using dummy scans (8 s). Motion correction was performed using FSL MCFLIRT. FSL BET was used for brain extraction (fractional intensity = 0.25, threshold gradient = 0.22, neck and bias-field correction). Images were spatially smoothed with 5 mm FWHM Gaussian kernel using *fslmaths*. Three-dimensional 3DMPRAGE anatomical images were used to register both the EPI BOLD and the MREG data into 4 mm MNI space prior to group ICA using FLIRT (Jenkinson and Smith, 2001; Jenkinson et al., 2002). FILM pre-whitening and data smoothness estimation was produced automatically by FSL MELODIC (Woolrich et al., 2001; Beckmann et al., 2006). FILM implements a robust method of correcting for auto-correlation in fMRI time-series which is (theoretically) independent of TR duration (Woolrich et al., 2001; Smith et al., 2004; Demetriou et al., 2018).

The MREG (TR = 0.1 s) data was downsampled to different sTR settings from TR 0.1 s to 0.3, 0.5, 1.0, 1.5, 1.8, 2.2, and 3.0 s, respectively by taking every n^th^ [n = 3, 5, 10, 15, 18, 22, 30] sample from every voxel timeseries. In addition, an interleaved variant of the MREG data were computed by taking every 1,3,5…21; 2,4,6…22th axial slice (hence the INT sTR = 2.2 s) to emulate the interleaved EPI data gathering for comparing single shot MREG trajectory downsampled at 2.2 s sTR data and conventional gradient recalled EPI (TR = 2.15 s). MATLAB was used for MREG data downsampling and interleaving. Total of 100 datasets were obtained after processing (10 sTR settings for 10 subjects).

As one of the focuses was to assess physiological signal aliasing effects, we retained the physiological pulsations in the data as much as possible. Therefore, cerebrospinal fluid (CSF) and white matter were not regressed out from the datasets like they often are in functional connectivity analyses. Global signal was analyzed but not regressed, as the benefit of its regression is still under debate (Murphy and Fox, 2017). Furthermore, the datasets were not de-spiked, since there is no clear consensus yet what kind of de-spiking is advisable to apply to ultra-short TR fMRI data, especially since most aggressive de-spiking (AFNI *3dDespike -NEW25*) removes some of the physiological pulsations from the data (Raitamaa et al., 2018).

### Time and Spatial Domain Analysis (ICA, CV/tSNR, DPARSF)

Group PICA was computed for all 10 sTR settings. For every group ICA run, 70 independent components were calculated using FSL MELODIC in default setting (Kiviniemi et al., 2009). FSL function *fslcc* was used to calculate correlation values between different PICA components calculated for every sTR and 42 resting state network templates defined earlier (Kiviniemi et al., 2009; Abou-Elseoud et al., 2010). Default mode network (DMN) posterior cingulate cortex (PCC), cerebral artery, visual, auditory, motor and ventral attention network components were selected as interesting reference components that were visualized to show the effect of changing sTR (Beckmann et al., 2005; Kiviniemi et al., 2009; Smith et al., 2009).

CV is a standardized measure used in e.g., engineering and physics, which describes the variability of a dataset compared to its mean. CV was used as a metric for the variation of physiological fluctuations in the signal. Recently, the CV of BOLD signal (CV_BOLD_) has been shown to be altered by the disease processes (Makedonov et al., 2016; Tuovinen et al., 2017; Kananen et al., 2018). CV was calculated for each subject and the calculation was carried out for every voxel timeseries:

$$\begin{array}{l} CV=\frac{\sigma }{\mu } \end{array}$$

where μ is the standard deviation, and σ is the mean of the voxel timeseries. Mean images of the resulting CV maps were computed. For statistical analyses, PCC region of interest (ROI) values were computed for every sTR individually and compared to mean reference (TR = 0.1 s) map. In addition, temporal signal-to-noise ratio (tSNR) values were computed for each sTR using white matter (WM) and gray matter (GM) ROIs. The ROI areas were obtained via FSL atlas tools using a probabilistic threshold value of 50. A mean value from both WM and GM were calculated from each subject.

### DPARSF (FC, ReHo, ALFF/fALFF)

For each FSL pre-processed dataset, seed-based functional connectivity (FC), regional homogeneity (ReHo), and amplitude and fractional amplitude of low frequency fluctuations (ALFF/fALFF) were calculated using Data Processing Assistant for Resting-State fMRI (DPARSF) V4.3_171210 MATLAB software package (Chao-Gan and Yu-Feng, 2010). The processing steps in DPARSF are described for each analysis method separately below. Global signal regression or white matter/CSF signal regressions were not used.

FC calculates seed-based correlation values between mean timeseries of a selected ROI area and the rest of the brain voxel timeseries. Six seed areas (5 mm spherical ROI) were selected for the analyses: DMN PCC (0 −53 26 mm in MNI), cerebral artery (0 32 16 mm in MNI), visual (2 −86 16 mm in MNI), auditory (50 −2 −8 mm in MNI), motor (2 −18 60 mm in MNI) and ventral attention network (-46 18 32 mm in MNI). Mean FC maps for each sTR and ROI were computed to see whether the changing of sTR influence the connectivity. For statistical analyses, correlation coefficient values were calculated between the mean ROIs (TR = 0.1 s) and other sTR settings individually using FSL function fslcc. Additionally, sTR 2.2 s (INT) and sTR 2.2 s were compared to test the effect of interleaving in MREG data.

ReHo measures the degree of regional synchronization of neighboring areas by calculating Kendall's coefficient of concordance (KCC) from the timeseries of every voxel and compares the neighboring voxels (Kendall and Gibbons, 1990; Zang et al., 2004). Cluster size of 27 voxels was used. Spatial smoothing (FWHM: kernel size [4 4 4]) was applied after ReHo calculations. After individual ReHo computations, mean spatial maps were calculated. Furthermore, ReHo KCC values were computed from the mean PCC ROI for all sTR settings.

### Frequency Domain Analysis

Fast Fourier transformation (FFT) amplitude spectra from a single subject arterial region ([−2 30 16] mm in MNI) (Figure 1) and global image signal, venous (high respiratory power) and arterial (high cardiac power) amplitude spectra were analyzed to observe the impact of sTR in different physiological frequency bands. Additionally, normal distributions curves (bin size 5) of histogram of demeaned global signal in every sTR were computed.

To examine, whether the change in sTR affects the VLF content, ALFF and fALFF were computed. ALFF calculates the sum of spontaneous low frequency activations of a selected frequency band (Zang et al., 2007). FALFF was used to estimate the ratio of amplitude spectrum of VLF in comparison to the whole FFT spectrum (Zou et al., 2008). Frequency band of 0.01–0.1 Hz was selected for the computations.

Furthermore, we studied how the mean FFT power of VLF (0.01–0.1 Hz), respiratory (0.12–0.35 Hz) and cardiac (0.9–1.3 Hz) pulsations occur in different sTR settings. The respiratory and cardiac frequency bands were chosen based on group-level minimum and maximum frequencies observed from the physiological recordings and datasets so that the band is as limited narrow as possible while still encompassing the cardiorespiratory frequencies of each subject. The same frequency bands were used for each subject. Each band was separated by a margin to minimize overlap.

Spectral maps of the frequency bands were calculated for each dataset using AFNI *3dPeriodogram*. The function outputs a power spectrum for each voxel timeseries. The frequency bins corresponding to VLF, respiratory and cardiac bands were collected from the periodogram datasets based on individual physiological monitoring. Furthermore, the collected bins were summed and finally mean of the sums was computed for each sTR. The cardiac bins were not calculated for sTRs > 0.5 s, as the slower sampling rates cannot extract the cardiac frequencies.

The effect of signal aliasing was estimated in two ways: (i) spatially, by correlating the mean cardiac and respiratory FFT power maps to individual respiratory and VLF maps for each sTR setting, respectively, (ii) power spectrally, by calculating the FFT power change in the respiratory and VLF bands in the right medial artery ROI ([46 −2 −8] mm in MNI) with different sTRs. Mean 0.1 s TR MREG datasets were used as baseline maps and compared how much the physiological power starts to overlap per given sTR.

### QPP Analysis

All datasets were bandpass filtered to VLF (0.01–0.1 Hz) band using AFNI *3dTproject*. We used a modified pattern finding algorithm to obtain quasi-periodic patterns (QPPs) and evaluated their changes in signal intensities and pulse propagations (Kiviniemi et al., 2016; Raitamaa et al., 2018). Estimation of timing and length of VLF pulse (length: 105–146 time points) for every subject was obtained from the VLF filtered global signal from TR 0.1 s. Length and timing were adjusted to the subsampled datasets. Subject-specific 4D QPP maps were created for every sTR. For the analyses, MATLAB *circshift* was used to ensure that QPP maps were in same phase.

QPP strength (i.e., how closely it resembled the template) of VLF wave for each sTR was quantified by correlation between the average QPP pulse and the VLF pulse at the last iteration of the QPP algorithm (Thompson et al., 2014). The mean correlation coefficients of VLF pulses from the last iteration were extracted from each subject and were compared as a function of sTR.

To measure the repeatability of the detected QPP maps, the spatial correlation of subject-specific average QPP maps of sTR > 0.1 s were compared to TR 0.1 s average QPP map using MATLAB *corrcoef*. For this, each sTR QPP map was interpolated to match the reference TR 0.1 s map.

For group mean images and videos, TR 0.1 s QPP maps were set to length of 150 time points and other sTRs lengths were adjusted accordingly. For every sTR, average group QPP maps were created.

### Statistical Tests

Paired sample *t*-tests (MATLAB *ttest*) were used for all statistical testing to compare the reference MREG data (TR = 0.1 s) with sTR results (Figures 3, 6, 7, 9A). Furthermore, sTR 2.2 s (INT) and sTR 2.2 s were compared to test the effect of interleaving in MREG data. In QPP repeatability analysis, linear fitting was used as a measure of statistical significance. Statistical significances are indicated as ^*^p < 0.05, ^**^p < 0.01, and ^***^p < 0.001.

## Results

As an example of the image quality of MREG 0.1 s TR data, Figure 1A illustrates a raw spatial image of one subject in three planes. An example of cardiovascular pulse timeseries (ROI MNI: [-2 30 16] in mm) from the anterior cerebral artery are presented in Figure 1B. Respective subsampling scheme is presented in Figure 1C, in which six examples of the sTR time series are shown to highlight the effect of changing sTR.

### Commonly Used Resting State Metrics (FC, ReHo, CV/tSNR, ICA)

**Figure 2:**
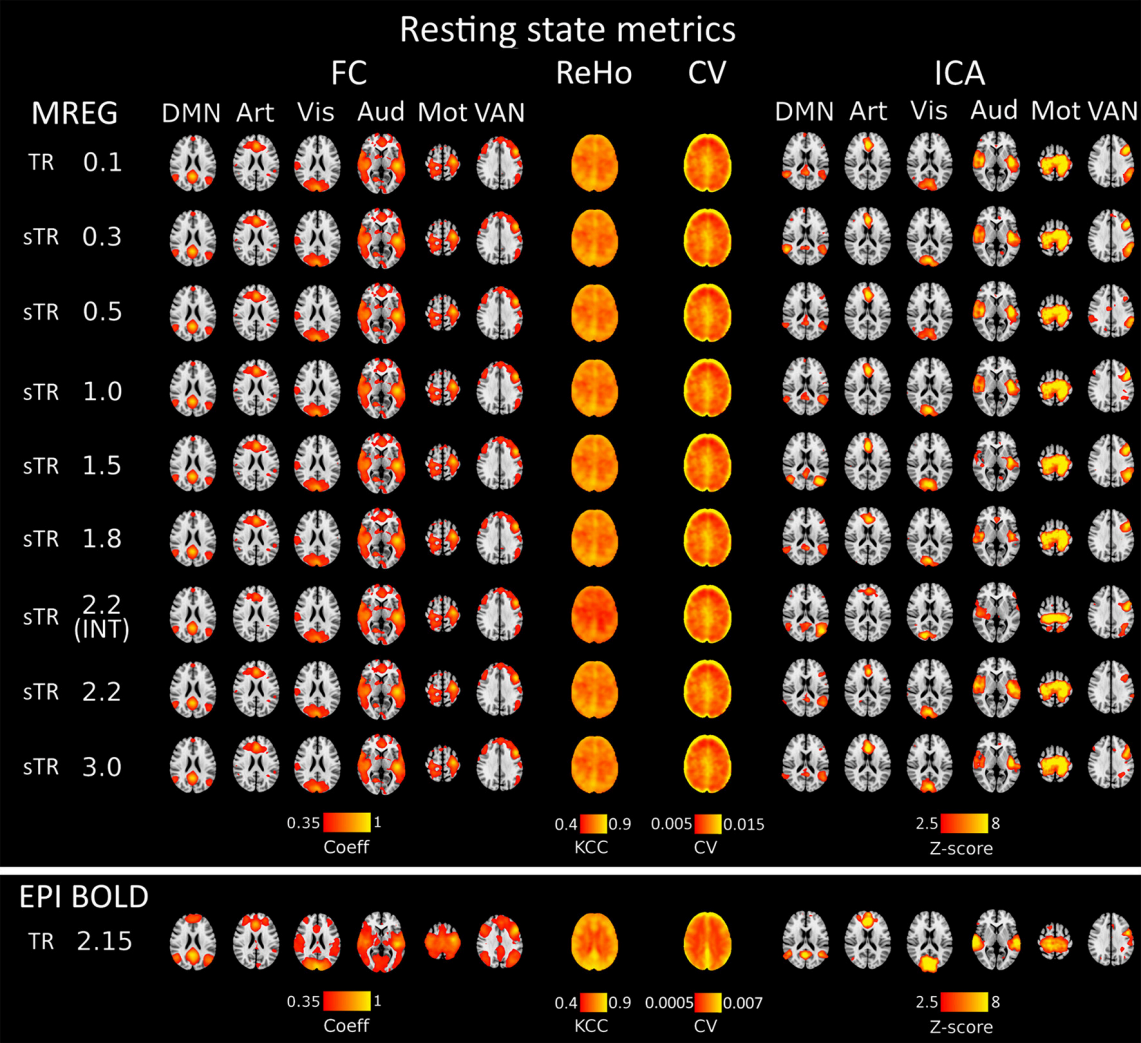
Different group-level resting state metrics with changing subsampled repetition times (sTR) including functional connectivity (FC), regional homogeneity (ReHo), coefficient of variation (CV) and independent component analysis (ICA). FC and ICA analyses comprise default mode network (DMN), artery (Art), visual (Vis), auditory (Aud), motor (Mot) and ventral attention network (VAN). For comparison, echo-planar imaging (EPI) maps below tends to show spatially more widespread FC maps and relatively similar ICA maps depending in the RSN.

Seed voxel correlation of FC analysis, ReHo and CV produced nearly identical results as a function of sampling rate. Visually, only small differences could be detected in the spatial functional connectivity measures in group level maps as a function of sampling rate in Figure 2.

**Figure 3:**
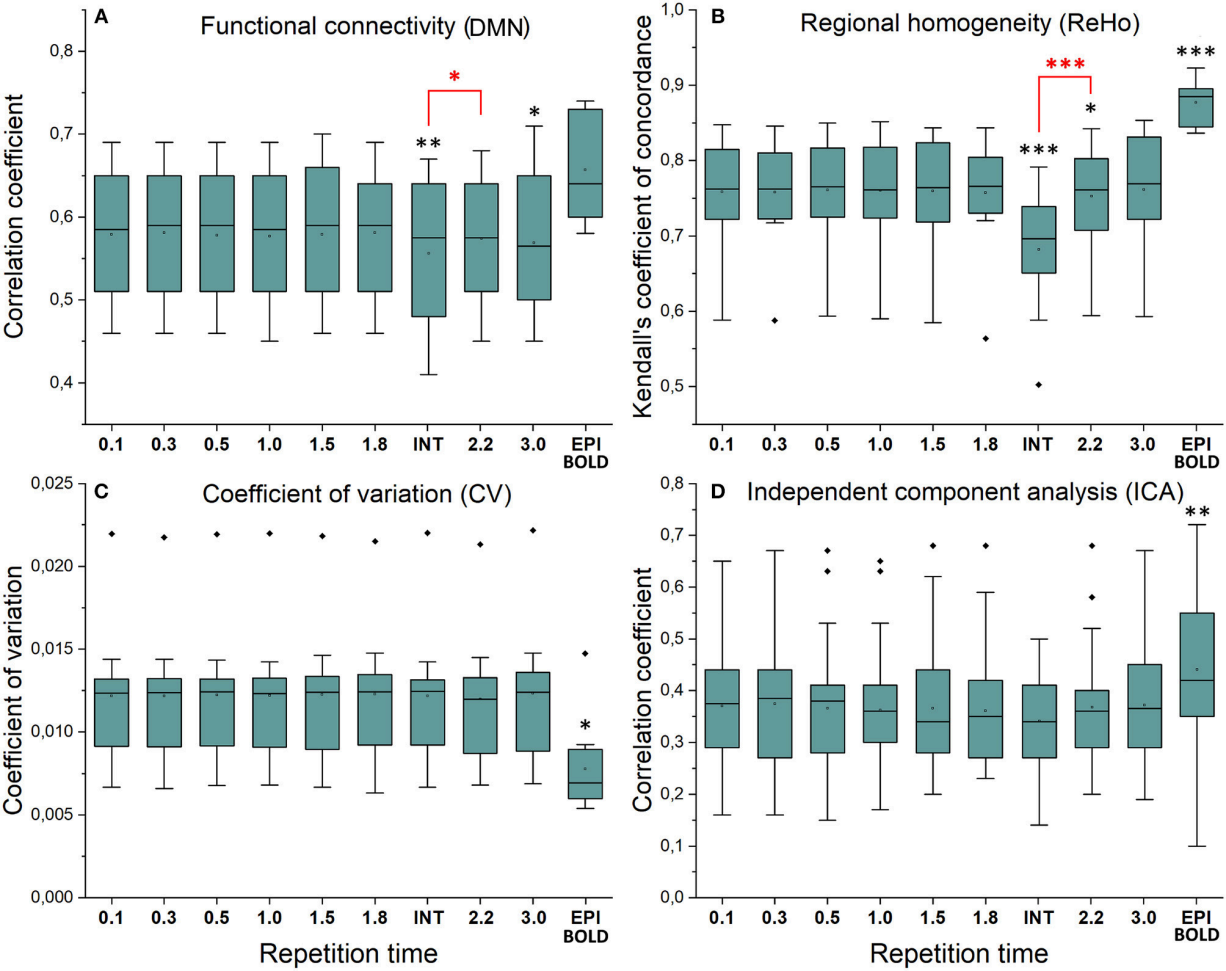
Resting state metrics in different subsampled repetition times (sTR) compared to reference maps (TR = 0.1 s). **(A)** Individual functional connectivity (FC) values from default mode network (DMN), posterior cingulate cortex (PCC ROI) correlated to reference FC map. **(B)** Regional homogeneity (ReHo) Kendall's coefficient of concordance (KCC) values from PCC ROI compared to mean reference map (TR = 0.1 s). **(C)** Coefficient of variation (CV) values from PCC ROI compared to mean reference map. **(D)** Correlation values between group probabilistic independent component analysis (PICA) components in different sTR settings and previously acquired 42 resting state network ICA templates. Significant differences between TR 0.1 and other sTR settings are marked with *p < 0.05, **p < 0.01, and ***p < 0.001. Outliers are marked with ♦.

Functional connectivity maps (Figure 2) showed nearly identical spatial results, which were also highly comparable to conventional 2.15 s TR EPI BOLD data or with interleaved MREG 2.2 s sTR data. The quantified connectivity measures presented no significant differences excluding INT (sTR = 2.2 s) which had significantly (*p* < 0.01) lower correlation coefficients compared to TR 0.1 s and compared to sTR 2.2 s (Figure 3A and Supplementary Figure 1). In addition, EPI scans had marginally elevated correlation coefficients compared to the MREG (TR = 0.1 s).

Similarly, ReHo were visually identical at different sampling rates (Figure 2). However, with interleaved sampling of 1,3,5…,2,4,6 of axial MREG slices were collected with a final 2.2 s sampling rate, the ReHo values decreased significantly (*p* < 0.001), when compared to both single shot k-space trajectory with TR 0.1 s and sTR 2.2 s (Figure 3B). Furthermore, conventional EPI showed significantly (*p* < 0.001) higher and sTR 2.2 lower (*p* < 0.05) values.

CV of the image data were nearly identical with respect to the altering sTR (Figure 2). The EPI BOLD data had significantly (*p* < 0.05) lower CVs compared to MREG (TR = 0.1 s), while downsampling or interleaved data gathering had no effect on the CV values. The same effect can be seen from the mean tSNR values from WM and GM ROIs where downsampling did not show any significant change but EPI BOLD values were significantly (*p* < 0.001) higher compared to MREG (Supplementary Figure 2).

ICA detected RSNs in all sTR settings, but there was some variability between the results (ICA in Figure 2). The conventional EPI BOLD and interleaved data showed similar results as well. In spatial consistency analysis of the 42 RSN template correlations, all sTR settings produced very similar results, except for EPI, which had significantly higher correlation values (*p* < 0.01).

### Mean Spectral Metrics

**Figure 4:**
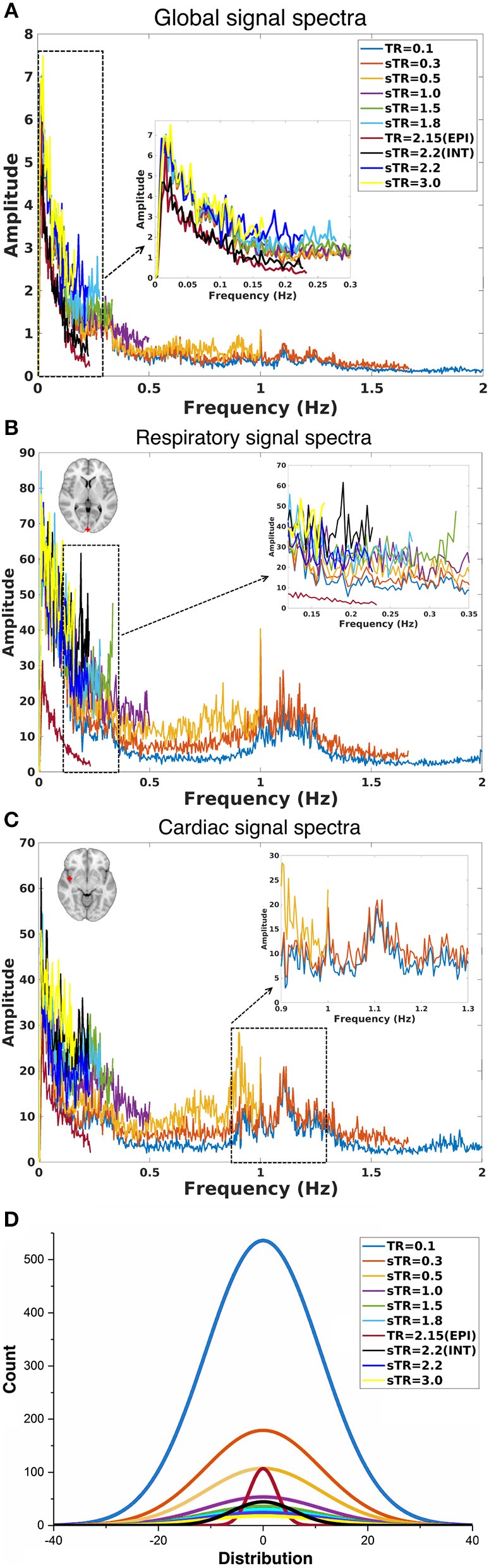
Group-level fast Fourier transform (FFT) amplitude and histogram distribution analyses in different subsampled repetition times (sTRs). **(A)** FFT amplitude spectra of global image signal where the 0.1 TR data is cut to 2 Hz from original 5 Hz. **(B)** FFT amplitude spectra of respiratory region of interest (sinus sagittal, ROI, [2 −98 4] mm in Montreal Neurological Institute (MNI) space). **(C)** FFT amplitude spectra of cardiac ROI (right medial artery) [46 −2 −8] mm in MNI. Due to differences in individual cardiorespiratory rates, several frequency peaks can be detected at the group level. **(D)** Mean normal distribution curves from demeaned global signal histograms show lowering and widening of the distributions as a function of sTR.

The group mean FFT amplitude spectra of global image signal, respiratory and cardiac signals are presented in Figure 4. In global signal spectra, the amplitude peaks were relatively higher in VLF band with increasing sTR. This might occur due to cardiorespiratory aliasing. The respiratory amplitude peaks from sagittal sinus and cardiac amplitude peaks from right middle cerebral artery presents multiple peaks in the spectra due to normal variations in respiratory and cardiac rates. Please notice, these peaks were lost as the sTR was increased >0.5 s. The same effect was observed in the arterial signal on a single subject level (Figure 1). EPI BOLD presented the lowest VLF amplitudes in all images.

The global signal distribution curves illustrated the differences in statistical power between sTR values and revealed an exponential decay in histogram counts and widening of the distributions per each sTR. Interestingly the 2.15 EPI BOLD distributions are highly similar in shape with MREG 0.1 s data but >5 times smaller.

### BOLD Signal Frequency Amplitude and Power Mapping

**Figure 5:**
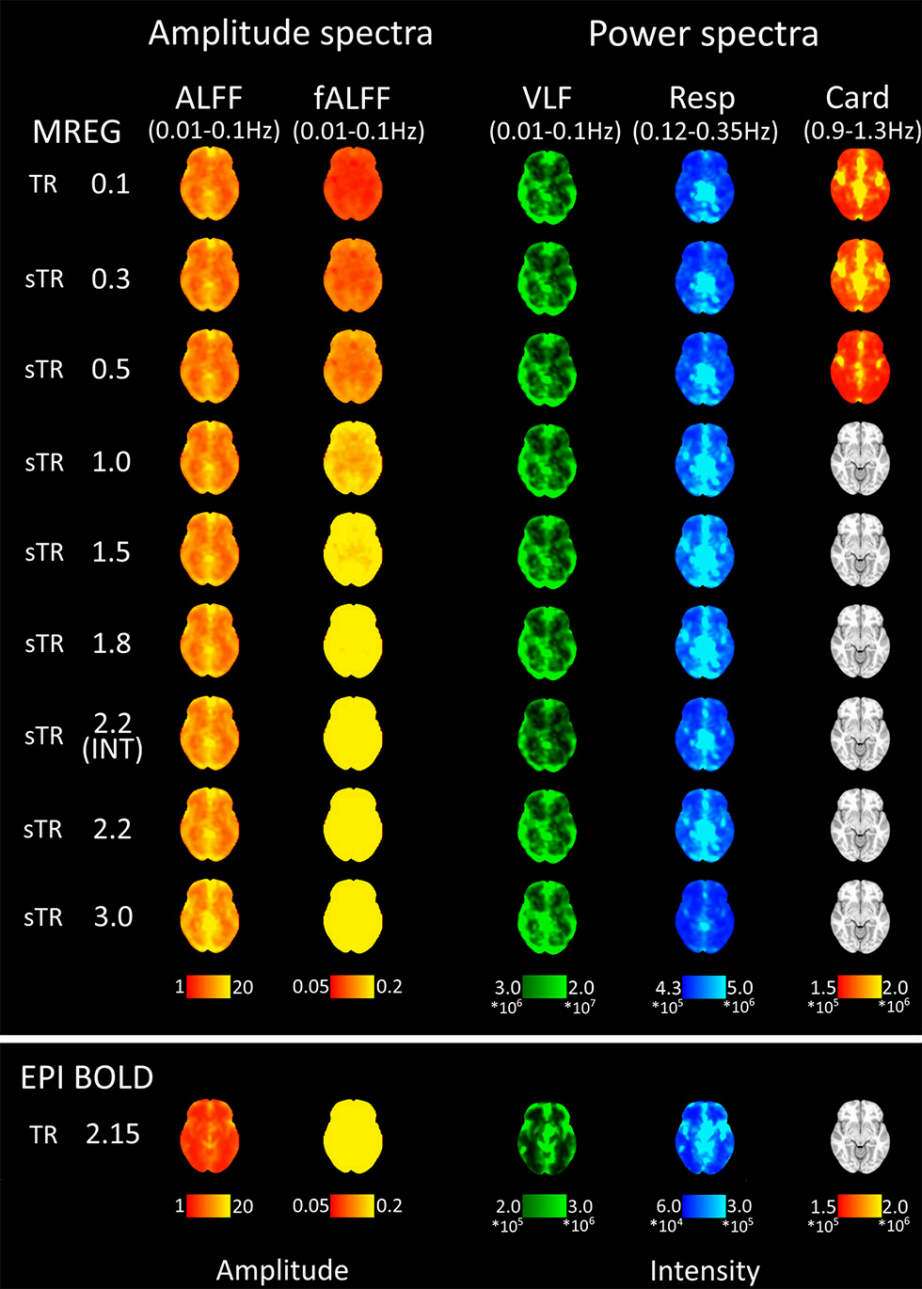
Mean fast Fourier transform (FFT) amplitude (ALFF/fALFF) and power (VLF, Resp, Card) encoding maps for each subsampled repetition times (sTRs). The cardiac power can be detected until 0.3 s and respiratory until 2.2 s sTR. However, the respiratory and cardiac power images started to overlap in sTR > 0.5 s, increasing to sTR 1–2 s. Very low frequency (VLF) power showed a subtle but steady increase with increasing sTR.

ALFF results indicated that the increasing sTR raised the VLF power of the images (Figure 5). Conventional EPI data showed lower ALFF values than MREG data, but the interleaving had no effect. FALFF results were most clearly affected by the increasing sTR (Figure 5). FALFF showed an increase over the sTR values which is due to the proportional increase of the lowest frequencies due to the reduction of the spectral coverage in higher sTR values.

**Figure 6:**
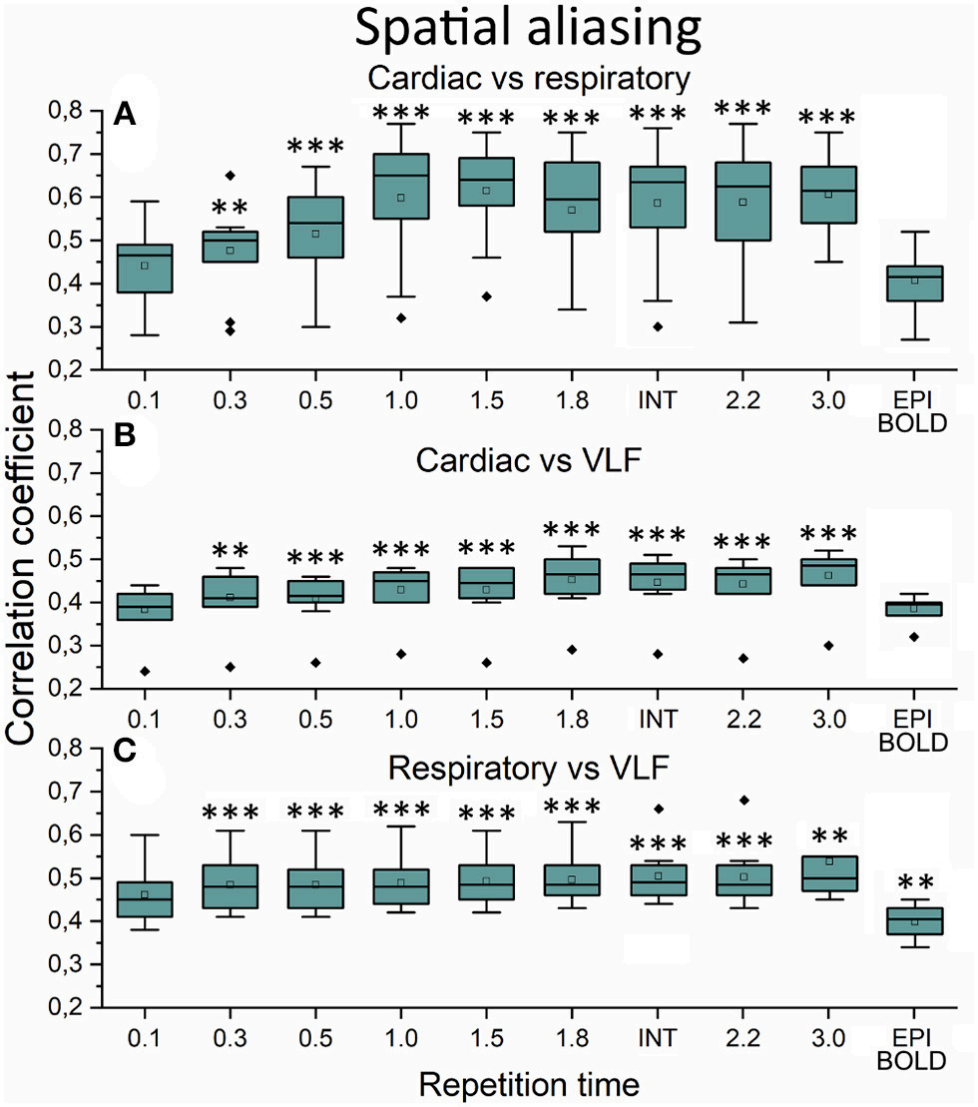
Spatial aliasing measure using spatial correlation values. **(A)** The mean MREG cardiac fast Fourier transform (FFT) power image of repetition time (TR = 0.1 s) correlated to individual respiratory power images as a function of sampled TR (sTR) and echo-planar imaging (EPI) scan. **(B)** Mean cardiac FFT image correlated to individual very low frequency (VLF) images. **(C)** Mean respiratory image correlated to individual VLF images. Significant differences between TR 0.1 s and other sTR settings are marked with **p < 0.01 and ***p < 0.001. Outliers are marked with ♦.

**Figure 7:**
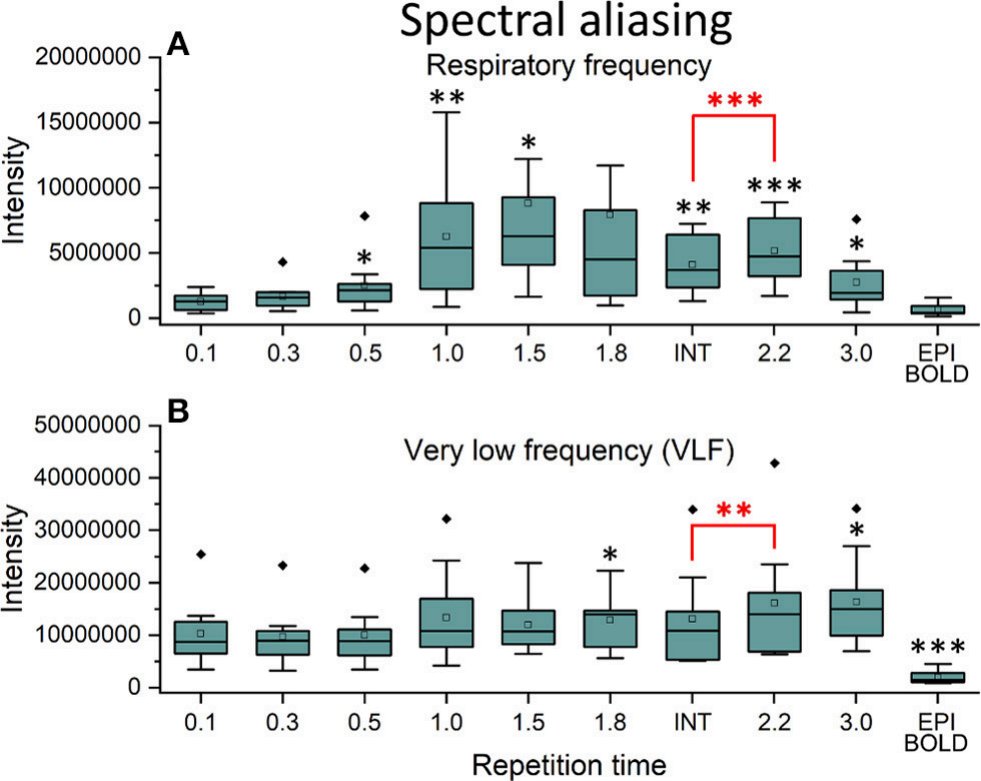
Cardiac aliasing measure using fast Fourier transform (FFT) power intensity values of arterial region of interest (ROI, [46 −2 −8] mm in Montreal Neurological Institute (MNI) space). **(A)** Intensity values of individual respiratory power images. **(B)** Intensity values of individual very low frequency (VLF) power images. Significant differences between repetition time (TR = 0.1 s) and other sampled TR (sTR) settings are marked with *p < 0.05, **p < 0.01, and ***p < 0.001. Outliers are marked with ♦.

Group mean FFT amplitude and power encoding maps (Figure 5) revealed how the cardiac, respiratory and VLF frequency intensities changed in different sTR settings. The cardiac power started to fade away in sTR >0.3 s and respiratory power above 2.2 s sTR. Furthermore, the cardiac power distributions started to overlap on top of respiratory frequency maps as the sTR is increased >0.5 s indicating aliasing, c.f. Figures 5–7. The conventional 2.15 s TR EPI measurement had notably lower power (please notice different scaling for EPI BOLD in Figure 5). Interleaved 2.2 s sTR power was also lower compared to 2.2 s sTR single shot trajectory in VLF power images, which agrees with ReHo and mean global signal amplitude spectral changes.

The aliasing was quantified both spatially and in frequency power analyses. This was evaluated by measuring how much different frequency maps start to resemble each other spatially as a function of sTR (Figure 6). In critically sampled 0.1 s TR MREG data the cardiac, respiratory and VLF frequencies had a low ~0.4 mean spatial correlation. For example, the major arteries seen in cardiac frequency maps were lacking from the respiratory maps, while the respiratory band dominated in posterior CSF spaces (Figure 5). However, the spatial correlation between cardiac and respiratory power maps became increasingly more similar (*p* < 0.001) and started to increase as a function of the sTR until 1.0 s, after which the similarity plateaus at 0.65 level until sTR 3.0 s (Figure 6A). Furthermore, the spatial correlation between cardiac (*p* < 0.001) and respiratory (*p* < 0.01) vs. VLF increased steadily until sTR 1.8 s.

In frequency power analysis of a right medial artery ROI ([46 −2 −8] mm in MNI) near insula, the image signal power from the arterial ROI started to increase significantly (*p* < 0.001) both in the respiratory and VLF power band in sTR >0.3 s as a sign of aliasing (Figure 7A). The results indicate that once the cardiac pulsation is no longer critically sampled, the power starts to become aliased as a “respiratory” and “VLF” power as a function of sTR increase. When correlating the mean respiratory maps to individual VLF maps, the aliasing effect could also be seen in VLF range as the correlation values increased significantly (*p* < 0.01) after 0.1 s TR and almost linearly as the sTR increased. However, the influence of the sTR had a clearly less steep effect by the increasing sTR in the VLF. The conventional 2.15 s BOLD images showed low spatial correlation since the cardiac power is very low to begin with and so the spatial correlation is also low.

### QPP Analysis of Spreading BOLD Waves

The power of cardiac pulsation could not be critically detected in sampling rates >0.3 s (Figures 3–5) and therefore we did not quantify differences in the detection of cardiac pulse propagation like we did earlier (Raitamaa et al., 2018). Also, the respiratory power suffered from marked aliasing in sTRs above 0.5 s (Figures 5–7). Therefore, we quantified the performance of different sTR in detecting VLF (0.01–0.1 Hz) propagating QPP BOLD waves.

**Figure 8:**
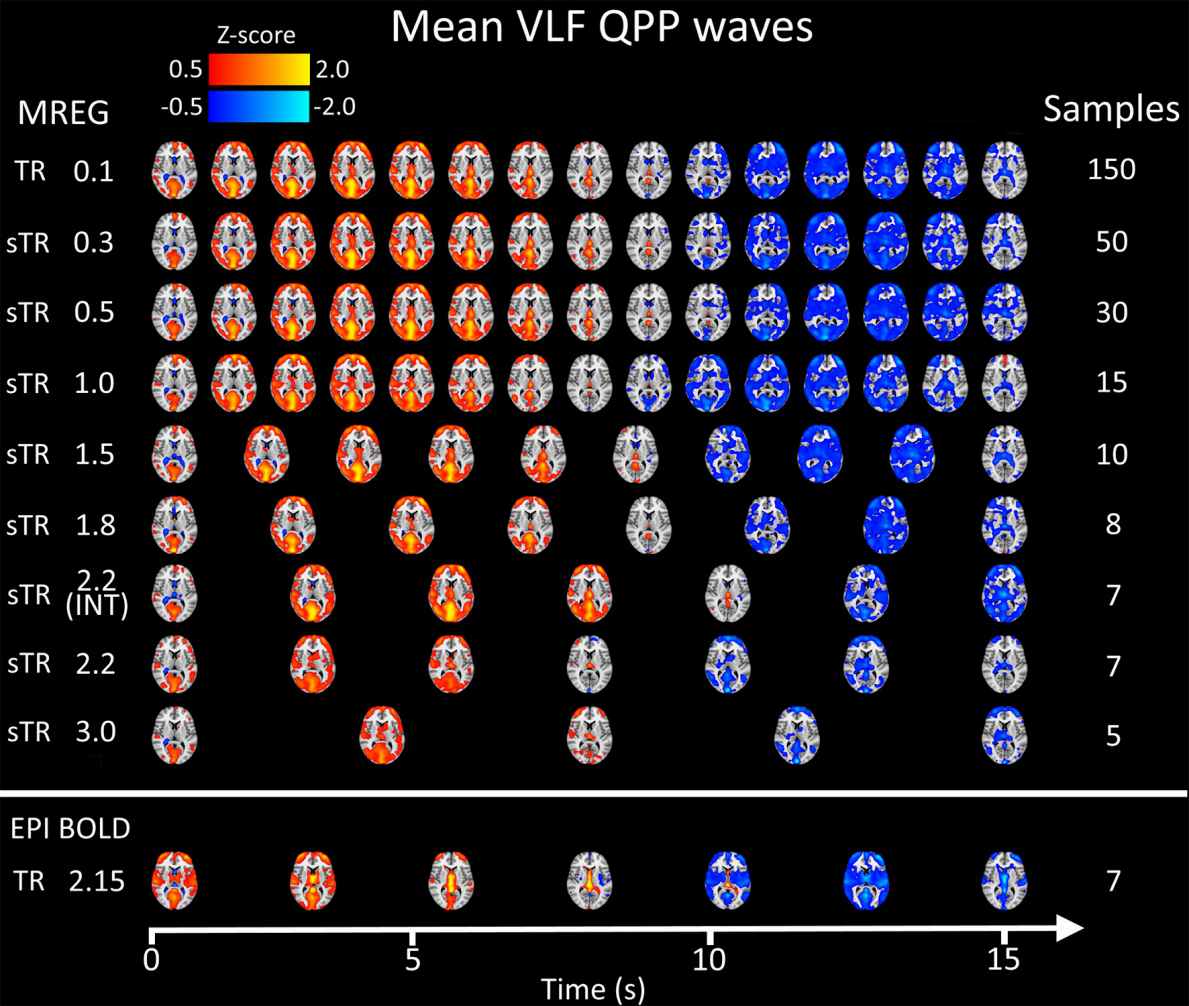
Group mean very low frequency (VLF) waves as a function of subsampled repetition time (sTR). Please notice that the first three rows are downsampled from 150, 50, and 30 to 15 images, respectively to fit the picture.

Figure 8 illustrates 15 s QPP waves captured with different sTR and how the spatiotemporal illustration of each wave changed as a function of sTR. The lowest sTR 0.1–0.5 s were down-sampled from 150, 50 and 30 to 15 images for illustration of the QPP wave spreading in axial plane. Videos of QPPs in TR of 0.1 s and 0.5, 1.0, 2.2 and 3.0 s are shown in Supplementary Material. As expected, much of the dynamics were lost as a function of increasing sTR.

**Figure 9:**
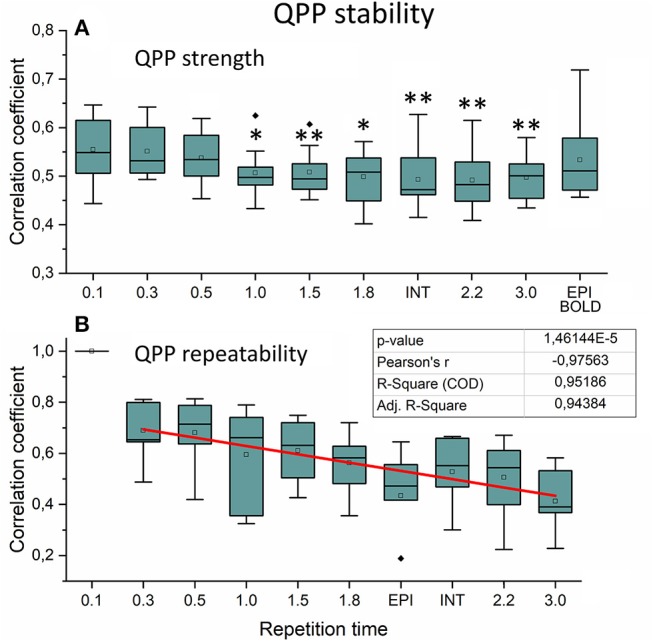
Very low frequency (VLF) quasi-periodic pattern (QPP) stability measurements. **(A)** Intra-individual QPP strength as a function of subsampled repetition time (sTR). Significant differences between repetition time TR 0.1 s and other sTR settings are marked with *p < 0.05 and **p < 0.01. **(B)** Inter-individual correlation coefficients of QPP wave repeatability as a function of sTR. Correlation coefficients decreased statistically significantly (*p* < 0.001) by decreasing slope of sTR. Outliers are marked with ♦.

We also quantified the QPP strength within each subject of the QPP VLF waves as a function of sTR (Figure 9A). The analysis revealed that 0.1 s TR data had highest spatial correlation of the detected VLF waves on average despite the largest number of brain volumes (i.e., 150 vs. 5 volumes between highest and lowest TR). There is a linear trend where the intra-individual detection accuracy of the detected VLF QPP waves gave lower (0.01 < *p* < 0.05) values as a function in sTR > 0.3 s excluding EPI.

When comparing how similar QPP waves were detected between subjects, the 0.1 s MREG data was used as a reference due to highest VLF wave detection accuracy. Each sTR QPP map was interpolated to correspond the 0.1 s MREG time points. On average, the detected QPP waves became linearly less correlated as a function of sTR (Figure 9B). Conventional EPI had less robustly detected QPP waves. Interleaved timing of data acquisition showed no effect on the wave detection accuracy nor on repeatability of the detected QPPs.

## Discussion

The study analyzed the effect of 3D image sampling rate on most popular fMRI metrics used. The time and spatial resting state fMRI metrics (FC, CV/tSNR, spatial ICA) were not markedly affected by sTR. In frequency domain analysis, the aliasing of the cardiorespiratory power seemed to increase signal power as a function of the sTR (sTR > 0.3 s). Importantly, aliasing effects occur mostly between cardiac and respiratory power. The VLF power increased also significantly as a function of sTR due to aliasing but the power of aliasing was smaller. In dynamic QPP analyses, shorter sTRs seemed to produce more stable results.

Also, to our surprise, the effect of sampling rate on non-dynamic rs-fMRI measures was not as strong as hypothesized. Most of the measures stayed stable within the range of sTR. Golestani and co-workers found surprisingly minimal effect on sampling rate on ReHo, ALFF and FC results that are in full agreement with our results (Golestani et al., 2017). Several researchers have found that higher sampling rate appears to be beneficial for resting state fMRI measures, especially for ICA/dual regression and our results also agree on this (Smith et al., 2013; Preibisch et al., 2015; Golestani et al., 2017; Demetriou et al., 2018.

Compared to MREG in general, the interleaved EPI BOLD had statistically significantly (*p* < 0.001) increased ReHo and tSNR. However, the f/ALFF, global signal amplitude spectrum baseline and signal variance as measured with CV are reduced in EPI compared to MREG. Interleaved MREG variant (INT sTR 2.2 s) also induced significantly (*p* < 0.001) lower ReHo compared to both TR 0.1 and to single shot variant (sTR 2.2 s). The conventional interleaving introduces TR/2 delay and interleaved multislab scanning introduces TR/2xN delay between subsequent scans, where N is the number of slabs. This introduces delays between neighboring voxels in longitudinal z-direction and alters regional similarity and frequency measures due to discontinuous sampling of the propagating cardiorespiratory pulses that traverse the brain repeatedly in addition to VLF pulses (Kiviniemi et al., 2016).

### Aliasing in Cortical Connectivity Analysis

The current data illustrates that while the aliasing of cardiorespiratory pulsations is a significant factor in frequency domain, the currently used time and spatial domain analyses tools perform well in detecting robust resting state connectivity. In summary, the stationary spatial and temporal connectivity measures, whether local or long distance, were not significantly affected by changes in sTR. However, interleaved slice acquisition seems to affect some of these measures.

This study confirms the information from reduced data length analytics (Bright and Murphy, 2015) and is consistent with coactivation pattern (CAP) analysis (Liu and Duyn, 2013), where the information on spatial functional connectivity of regions can be depicted even in one single brain volume. However, the CAP analysis for instance, requires individual voxel level thresholding with the time domain signal mean/std.

ICA performs best with large data distributions and therefore conventionally spatial ICA is preferred over temporal ICA, since it offers larger distributions (Calhoun et al., 2001; Kiviniemi et al., 2003; Beckmann et al., 2005). Spatial ICA also uses BOLD signal's temporal variance which induces the non-Gaussian changes in the signal distributions, by which the statistical independence is inferred (Calhoun et al., 2001; Kiviniemi et al., 2003; Beckmann et al., 2005). The clear advantage of the short TR can be seen in Figure 4, where 0.1 s TR overall signal distribution histogram has almost 5 times higher and substantially larger distribution compared to other distributions. This is the basis of the statistical power advantage already depicted by several groups for using short TR measurements (Smith et al., 2013; Preibisch et al., 2015; Golestani et al., 2017; Demetriou et al., 2018). Furthermore, in the case of combined spatiotemporal ICA or temporal ICA alone approaches, the 0.1 s data most likely outperforms the slower sTRs simply due to statistically more valid signal histograms (Figure 4D).

The physiological pulses themselves modulate the BOLD signal (Birn et al., 2006, 2008; Chang and Glover, 2010; Chang et al., 2013a). Recently, ultra-fast MREG data was able to illustrate how the cardiac and respiratory pulsations propagate in repeated waves over the whole brain, giving rise to physiologically driven and modulated variance in the data (Kiviniemi et al., 2016; Raitamaa et al., 2018). However, most of the cardiorespiratory power is centered within central areas near CSF ventricles (respiratory) and major arteries and veins (cardiac). The VLF power dominance in the brain cortex seems to enable robust connectivity measurement despite imminent cardiorespiratory aliasing especially in sTRs 1–2 s.

The physiological signal sources need to be separated from the functional signals before any realistic interpretations of the neurovascular task or connectivity data can be drawn. Through the years, source separation tools such as the ICA have been developed to offer robust detection of functionally connected regions or constellations of regions, such as resting state networks (Calhoun et al., 2001; Kiviniemi et al., 2003; Beckmann et al., 2005; Griffanti et al., 2015; Vidaurre et al., 2017). However, in this respect also other measures, such as FC and CV are highly reproducible over a range of sTRs. Therefore, even though there is significant aliasing, especially in areas near medial cerebral artery and sagittal sinus neighborhood, the dominance in VLF induces robust functional connectivity in the cortex.

BOLD signal stability is often measured as tSNR or CV which is the inverse of tSNR. In this study, we calculated both. In terms of signal observations of hemodynamically convolved neuronal activation response, in our sampling scheme the CV or tSNR do not change as we only downsample the MREG data instead of scanning using different TRs. However, compared to EPI BOLD the CV values were significantly higher (Figures 2, 3) and tSNR values significantly lower (Supplementary Figure 2) which could be partially caused by higher flip angle and slightly shorter TE in EPI BOLD. In spite of all, both sequences have relatively long TEs, which makes them T2^*^ -weighted.

Recent studies indicate how the CV reflects also physiological pulsation changes, rather than neuronally driven alterations. Furthermore, the CV has recently been shown to have high sensitivity to pathological condition even at individual level (Makedonov et al., 2016; Tuovinen et al., 2017, 2018). Our preliminary experience on disease related BOLD signal noise metrics suggests that at 1.8 s TR the BOLD signal CV can also be highly sensitive to pathological conditions (Tuovinen et al., 2018). Data from 0.1 s MREG CV measures seem to be at least as sensitive in intractable epileptic patient data (Kananen et al., 2018). This is in line with the CV results that do not alter as a function of TR but can be sensitive to differences in sequence parameters and k-space trajectory and other technical issues, c.f. Figure 2.

### Aliasing of Physiological Pulsations in Central Areas

The FFT frequency power maps illustrate that the cardiac 1 Hz power is mainly detectable in sTR < 0.3 s. In the most critically sampled data, the 1 Hz cardiovascular signal pulses most prominently in the paravascular space in areas near major cerebral arteries and somewhat in the major venous sinuses. From the periarterial areas the cardiovascular pulses are convected into the CSF ventricles, centerline parasagittal CSF spaces, c.f. Figure 5. The respiratory power is nearly absent from the periarterial spaces and dominates more on the posterior and cortical perivenous structures and is relatively stronger in the posterior central CSF spaces. The VLF power tends to align mostly along the cortical gray matter without strong overlap with the midline parasagittal area.

Above 0.3 s sTR, the cardiac power became mostly aliased over respiratory power in central brain bordering CSF spaces. Thus, all critical analysis of respiratory power changes needs to be performed with data < 0.5 s TR. The most prominent aliasing occurred in sTRs between 1 and 2 s, where the respiration is still sampled critically but the cardiac is aliased over it; they become strongly mixed into sTR 1–2 s signal. This type of cardiorespiratory aliasing however seems to affect the measuring of FC on cortical structures minimally (Figures 2–3), since the connectivity occurs dominantly in VLF frequencies (< 0.1 Hz) that dominate in the cortex to begin with. Also, the effects of aliasing as a function of sTR on the VLF FFT power are less severe around sTR 1–2 s but increase further in sTRs >2.0 s (Figures 2, 5, 6).

The power of FFT amplitude spectra increased in VLF range as a function of sTR, which is a sign of cardiorespiratory aliasing (Kiviniemi et al., 2005; De Luca et al., 2006). The effect was highest in sTR 3.0 s. This reduces sensitivity to changes in physiological pulsations and furthermore does no longer have the capability to differentiate neither cardiorespiratory nor even different VLF fluctuation peaks as different sources. Furthermore, physiological pulsations seen in 0.1 s TR could not be detected on a group level global or individual voxel signal above 0.3 s sampling rate. Also, VLF power peak features became undetectable with vanishing power of the increased sTR.

Importantly, the f ^−α^ FFT amplitude spectrum curve became affected by the highest sTRs (Figure 4). This has an inevitable effect on measures of signal stationary metrics like Hurst exponent (H) (Bullmore et al., 2001; Wink et al., 2008), fractal dimension (Df) (Kiviniemi et al., 2005; Kiviniemi, 2008) since the power spectral intensity f(I) = f ^−α^, where Df = (3-α)/2 and *Df* = 2-*H*. The f/ALFF as well as f ^−α^ metrics may then also be sensitive to physiological pulsations. In other words, results based on comparing patients with controls may suffer from cardiorespiratory differences between groups. To avoid these factors, physiological signals should be measured and FFT power spectral analyses should be performed on as short TR data as possible. This is our recommendation for future studies.

### Dynamic Connectivity and Effect of TR

The recent discoveries of dynamic functional connectivity analytics of fMRI data show that there are marked changes over time (Hutchinson et al., 2012). Wavelet analyses and time windowed approaches have suggested that the connectivity of regions varies markedly over time (Chang and Glover, 2010; Kiviniemi et al., 2011; Smith et al., 2012; Liu and Duyn, 2013). Targeted averaging algorithms can detect quasi-periodic very low frequency BOLD signal pulses that travel over the RSN and connectivity gradient patterns (Majeed et al., 2011; Pan et al., 2013; Keilholz, 2014; Thompson et al., 2014).

In this study we evaluated the VLF QPP maps to quantify effects of sTR on dynamic BOLD connectivity metrics. First, the FFT analysis indicated that the faster cardiorespiratory pulsations could not be even evaluated as a function of sTR: the cardiac power is not visible above 0.5 s sTR and furthermore the cardiac power aliases over respiratory power. Secondly, the VLF pulse analysis indicated that compared to the most critically sampled 0.1 s TR data, both the QPP strength (i.e., spatial similarity of waves) and inter-individual repeatability of the QPPs become linearly reduced with increasing sTR. In other words, the QPP analysis significantly lost its accuracy as a function of sTR. Taken together, the analysis of dynamic and physiological pulsations benefits when performed on data with TRs < 0.3 s.

As with most of the dynamic connectivity metrics, the problem has been the reducing statistical power of the analysis when shorter and shorter connectivity epochs have been attempted to be analyzed. Reducing dynamic analysis window length reduces also the number of samples and degrees of freedom for statistical inferences. Our results support the use of short TR in the evaluation of dynamic connectivity metrics as (a) for mathematical procedures, short TR gives more time points per analyzed time window, (b) it enables critical sampling and differentiation of physiological pulsations, (c) it avoids aliasing of the physiological signal over the targeted phenomena, (d) enables mapping of modulations of the pulsations, and e) it offers markedly more accurate signal distributions for statistical inferences, c.f. Figure 4D.

## Limitations and Strengths of the Study

The results may not be directly comparable due to multiple technical differences between interleaved EPI vs. MREG. However, despite the technical differences, the results in stationary connectivity are highly similar in a wide range of frequencies and RSNs. This study aimed to minimize confounding factors like intra-subject status changes (cardiorespiratory status, vigilance, mood, etc.) as well as technical factors (SNR, sequence parameters, sampling trajectory, scanner model, field strength) issues by using data from the same subjects scanned once with fast TR that was downsampled to higher sTRs. This enables the direct comparison of the effects of sampling rate only. EPI data was scanned for comparing the results to a more conventional technique.

However, the interleaved vs. single shot k-space sampling technique is not the only difference when comparing MREG and EPI data. As mentioned accurately by Golestani and co-workers, comparisons of long vs. short TR measurements need to be taken with a grain of physics, i.e., the EPI vs. MREG also have several other technical differences. In order to be fast, TR is very short in MREG which sensitizes the signal more to T1 inflow and steady state precession effects compared to conventional EPI BOLD (Liu, 2016). On the other hand, TE is relatively long in MREG which makes it T2^*^ -weighted as is EPI BOLD. The low flip angles 5° vs. 15° in MREG and EPI BOLD, respectively, reduces the sensitivity to physiological pulsations on both methods as well (Gonzalez-Castillo et al., 2011). Furthermore, the system noise changes linearly or even quadratically with the TR due to imperfections as the sequence is repeatedly run. All these effects need to be considered while discerning the differences between EPI vs. MREG results and again the direct comparison between them is rather difficult.

According to (Glover, 2012), the spiral readout has reduced sensitivity to motion, shortened readout times, improved signal recovery in most frontal and parietal brain regions, and exhibited blurring artifacts instead of ghosts or geometric distortion. MREG combines Spiral-in/out trajectory which further has diminished susceptibility-induced signal dropout and increased BOLD signal. The EPI readout trajectory is subject to ghosts from off-resonance and gradient imperfections and is intrinsically sensitive to cardiac-induced pulsatile motion from substantial first- and higher order moments of the gradient waveform near the k-space origin (Glover, 2012). So, the artifact and BOLD sensitivity profiles are also different between EPI and MREG. In summary, looking at the similarity of the resting state connectivity measures with different techniques, it seems that human brain connectivity is quite a stable phenomenon that can be measured robustly with technically quite different scanning approaches.

Earlier, the use of two different sampling rates scanned at subsequent scanning sessions have been used to exclude aliasing as a source of very low frequency fluctuations as a source of resting state functional connectivity (Beckmann et al., 2005; Kiviniemi et al., 2005). Theoretically, the previous data that used different scan sessions and so the physiological pulses were not identical between the TRs and therefore not directly comparable. However, in studies with different sampling rate in different scan, also there the results seem to be highly similar to our data (Smith et al., 2013; Golestani et al., 2017).

While attempting to quantify aliasing and dynamic metrics, we used the original 0.1 s TR MREG data as a reference. This may in theory involve a bias since the original MREG data can also detect modulations of cardiorespiratory pulse amplitudes and timing variations, as seen in Figure 1. The slower sTRs have no way of depicting these modulations and therefore a comparison of spatial connectivity and signal stability may be somewhat biased toward undermining the accuracy of the fast data, since it is also sensitivity to modulation effects.

## Conclusion

The overall the effect of sampling rate on most commonly used stationary rs-fMRI metrics is minimal. The dominance of the VLF power in gray matter overpowers aliasing effects and enables highly reproducible stationary connectivity results. The aliasing is most dominant between cardiac and respiratory pulsations in central structures near or within CSF spaces. Different technical imaging approaches (e.g., interleaved EPI vs. SOS MREG) yield differential connectivity metrics stemming from multiple spin acquisition differences. Despite these differences, the results from different techniques give fairly good spatial agreement of human brain connectivity. Interleaved scanning of slices seems to introduce inaccuracies in some analyses due to discontinuous sampling of physiological signals. The analysis of dynamic connectivity and frequency based physiological pulsation benefits most from faster scanning.

## Data Availability

The datasets generated for this study are available on request to the corresponding author.

## Author Contributions

VKi and VKo: designed the study; NH, VKi, VKo, and VR: collected the data; NH, LR, VKi, VKo: analyzed the data; NH, VKi, VKo, JaK, LR, HH, VB, TT, AR and JuK: wrote the manuscript.

### Conflict of Interest Statement

The authors declare that the research was conducted in the absence of any commercial or financial relationships that could be construed as a potential conflict of interest.

## Supplementary Material

Video 1 Mean very low frequency quasi-periodic patterns (VLF QPPs) for repetition time (TR = 0.1 s).

Video 2 Mean very low frequency quasi-periodic patterns (VLF QPPs) for subsampled repetition time (sTR=0.3 s).

Video 3 Mean very low frequency quasi-periodic patterns (VLF QPPs) for subsampled repetition time (sTR = 0.5 s).

Video 4 Mean very low frequency quasi-periodic patterns (VLF QPPs) for subsampled repetition time (sTR=1.0 s).

Video 5 Mean very low frequency quasi-periodic patterns (VLF QPPs) for subsampled repetition time (sTR=1.5 s).

Video 6 Mean very low frequency quasi-periodic patterns (VLF QPPs) for subsampled repetition time (sTR=1.8 s).

Video 7 Mean very low frequency quasi-periodic patterns (VLF QPPs) for subsampled repetition time (sTR=2.2 s).

Video 8 Mean very low frequency quasi-periodic patterns (VLF QPPs) for subsampled repetition time (sTR=3.0 s).

Video 9 Mean very low frequency quasi-periodic patterns (VLF QPPs) for conventional echo-planar imaging (EPI) acquisition with repetition time (TR=2.15 s).

Video 10 Mean very low frequency quasi-periodic patterns (VLF QPPs) for interleaved variant with subsampled repetition time (sTR=2.2 s).
