# Supplementary figures and images for: Sampling Rate Effects on Resting State fMRI Metrics
Source: Front Neurosci. 2019 Apr 2;13:279. doi: 10.3389/fnins.2019.00279 (PMC6454039; doi:10.3389/fnins.2019.00279)

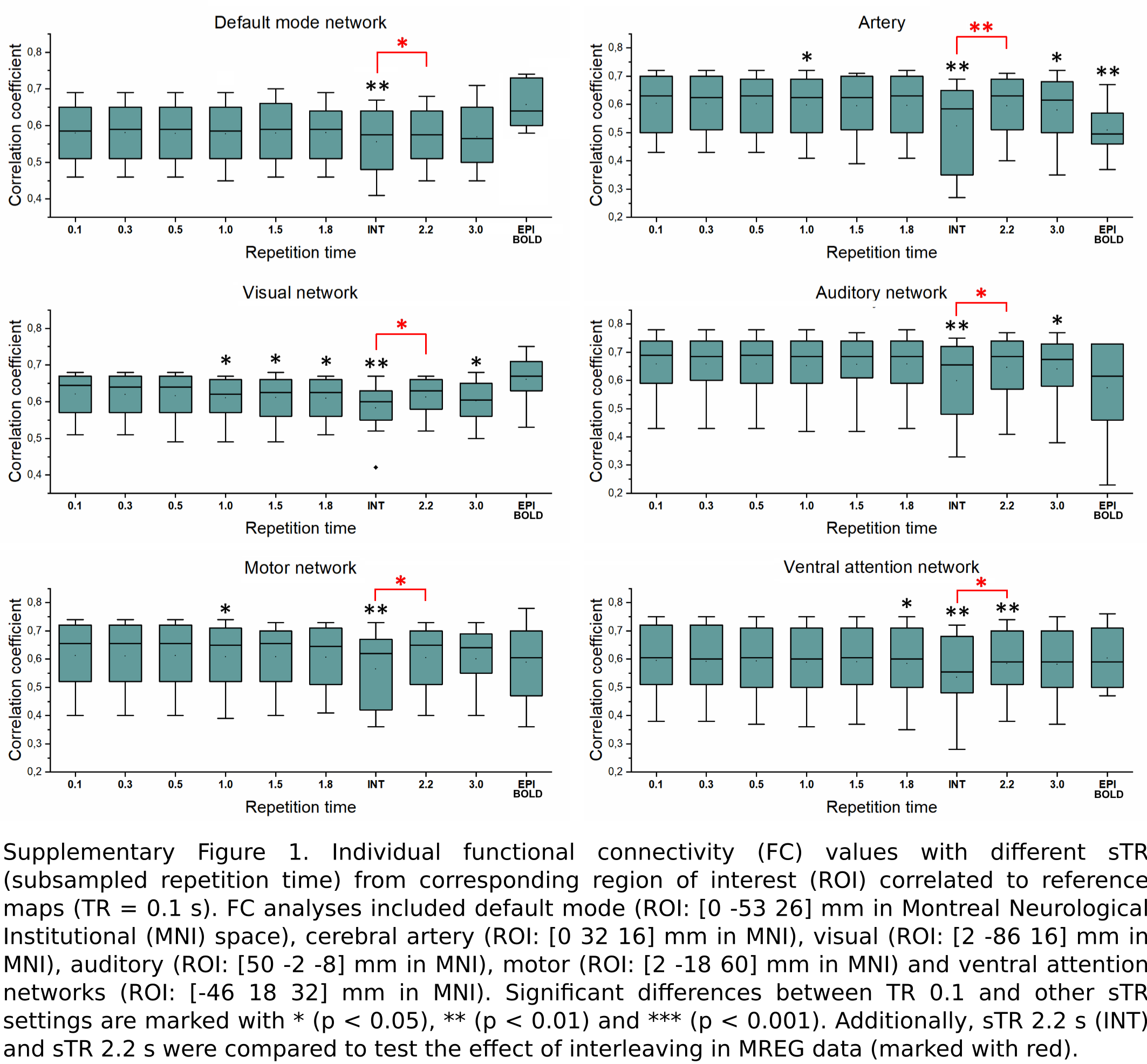

Supplement: Supplementary file 1 [file Image_1.TIF]

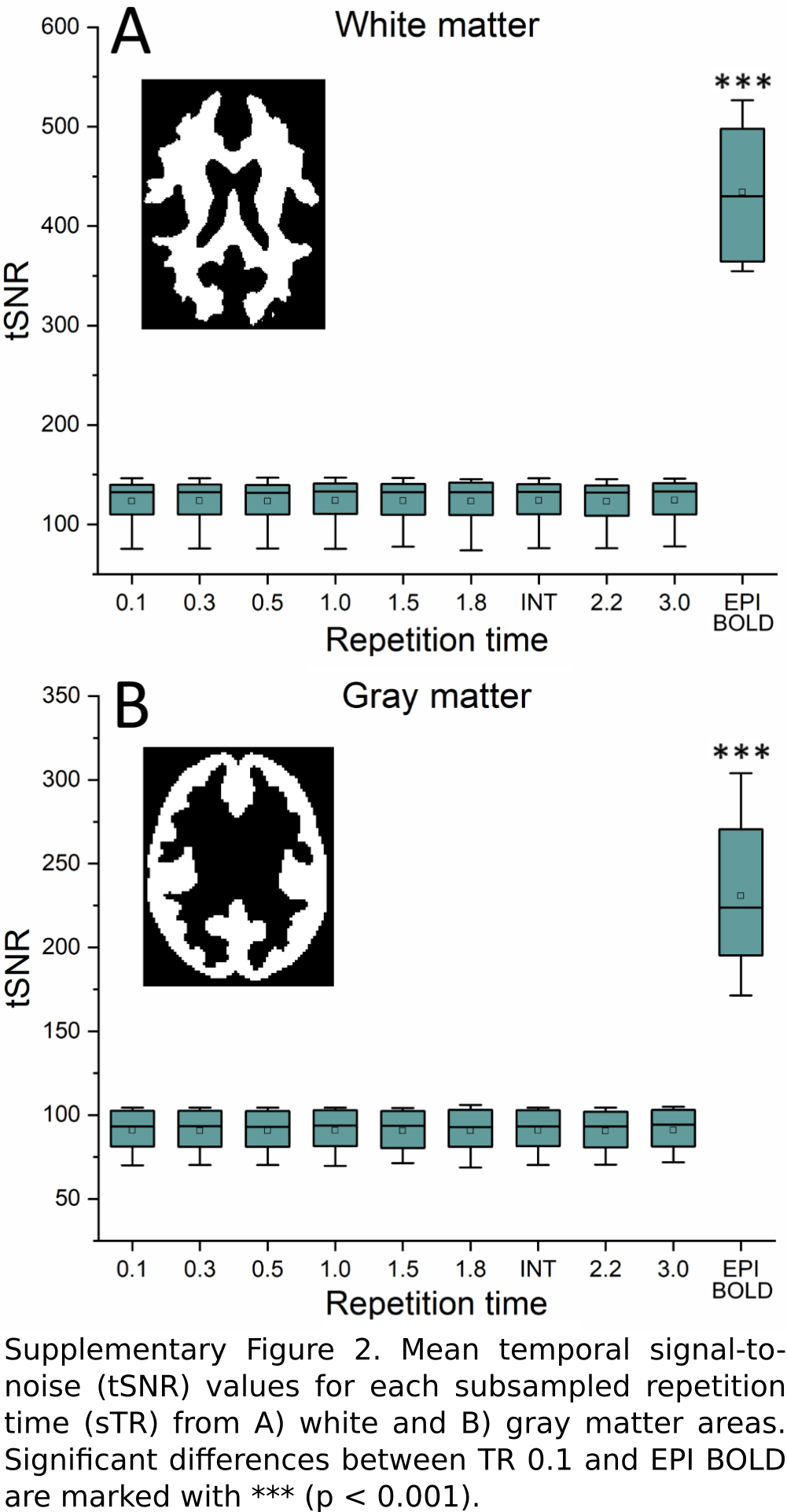

Supplement: Supplementary file 2 [file Image_2.TIF]
